# Design considerations for early-phase clinical trials of immune-oncology agents

**DOI:** 10.1186/s40425-018-0389-8

**Published:** 2018-08-22

**Authors:** Nolan A. Wages, Cody Chiuzan, Katherine S. Panageas

**Affiliations:** 1 Division of Translational Research & Applied Statistics, Department of Public Health Sciences, University of Virginia, P.O. Box 800717, Charlottesville, VA USA; 2 Department of Biostatistics, Mailman School of Public Health, Columbia University, New York, NY USA; 3 Department of Epidemiology & Biostatistics, Memorial Sloan Kettering Cancer Center, New York, NY USA

**Keywords:** Immunotherapy, Early-phase, Clinical trials

## Abstract

**Background:**

With numerous and fast approvals of different agents including immune checkpoint inhibitors, monoclonal antibodies, or chimeric antigen receptor (CAR) T-cell therapy, immunotherapy is now an established form of cancer treatment. These agents have demonstrated impressive clinical activity across many tumor types, but also revealed different toxicity profiles and mechanisms of action. The classic assumptions imposed by cytotoxic agents may no longer be applicable, requiring new strategies for dose selection and trial design.

**Description:**

This main goal of this article is to summarize and highlight main challenges of early-phase study design of immunotherapies from a statistical perspective. We compared the underlying toxicity and efficacy assumptions of cytotoxic versus immune-oncology agents, proposed novel endpoints to be included in the dose-selection process, and reviewed design considerations to be considered for early-phase trials. When available, references to software and/or web-based applications were also provided to ease the implementation. Throughout the paper, concrete examples from completed (pembrolizumab, nivolumab) or ongoing trials were used to motivate the main ideas including recommendation of alternative designs.

**Conclusion:**

Further advances in the effectiveness of cancer immunotherapies will require new approaches that include redefining the optimal dose to be carried forward in later phases, incorporating additional endpoints in the dose selection process (PK, PD, immune-based biomarkers), developing personalized biomarker profiles, or testing drug combination therapies to improve efficacy and reduce toxicity.

## Background

The advent of the breakthrough therapy designation for experimental drugs by the Food and Drug Administration (FDA), initiated as part of the FDA Safety and Innovation Act (FDASIA) in July 2012, has taken oncology drug development in a new direction. A therapy receives this FDA designation if it is “one which is intended alone or in combination to treat a serious or life-threatening disease or condition, and for which preliminary clinical evidence indicates the drug may demonstrate substantial improvement over existing therapies on one or more clinically significant endpoints” [1, 2]. Such a designation from the FDA accelerates the review and development process of the new therapy, prompting regular communication between the FDA and the sponsor to guide the development of promising agents. Since the FDASIA was signed into law, there have been several accelerated approvals of cancer drugs, particularly immune-oncology agents. For example, the immune checkpoint inhibitor pembrolizumab was FDA-approved for the treatment of melanoma in December 2014, three months after publication of the Phase I data [3]. Approval was based on the results from a dose-comparing, activity-estimating cohort within a multicenter open-label randomized phase 1b clinical trial. The major efficacy endpoint was confirmed overall response rate. As a condition of this accelerated approval, Merck was required to conduct a multicenter randomized trial establishing the superiority of pembrolizumab over standard therapy to verify and describe its clinical benefit. These accelerated approvals have led to a substantial increase in the number of clinical trials testing immunotherapies. The efficacy exhibited by checkpoint inhibitors in melanoma patients [4–6] triggered further testing in other cancer subtypes such as non-small cell lung cancer [7, 8], renal cell cancer [9, 10], bladder cancer [11], and Hodgkin Lymphoma [12]. Other immune-oncology treatment strategies include monoclonal antibodies, cancer vaccines, and adoptive T-cell therapies such as chimeric antigen receptor (CAR)-modified T-cells.

The changing landscape of oncology drug development has resulted in a significant departure from the historical paradigm of a clinical trial design, especially in early development. Phase I trials have served as initial safety studies, with the main objective of identifying the maximum tolerated dose (MTD). The MTD is the highest dose among a range of predefined dose levels that satisfies some safety requirement. The underlying assumption driving the design of a Phase I trial is that both the risk of toxicity and the probability of clinical benefit increase with dose level; thus, the MTD represents the most promising dose for efficacy. The primary toxicity endpoint of interest is most often a binary one, defined in terms of the proportion of patients who experience a dose-limiting toxicity (DLT; yes/no), based on protocol-specific adverse event definitions. The definition of DLT will be defined at the design stage and will differ from trial to trial, depending on disease specification and agent being tested. It typically is characterized by a grade 3 or higher adverse event according to the National Cancer Institute (NCI) Common Terminology Criteria (CTCAE) in the first cycle of treatment. The primary objective is to locate the MTD, defined as highest dose that can be administered to and tolerated by an acceptable proportion of patients. The MTD is often the recommended Phase II doses (RP2D) under the assumption that higher doses are likely to be more effective.

While the traditional approach to Phase I clinical trial designs is adequate for testing cytotoxic agents (e.g., chemotherapy), immune-oncology agents have different assumptions and challenges, some of which may also be applicable to targeted therapies. Thus, trial designs and the corresponding endpoints need to be adapted to the specific agents being investigated [13, 14]. There is a growing need for the implementation of new study designs that address the clinical realities and statistical considerations arising from these new treatment paradigms. In this article, we discuss some of the statistical challenges that immune-oncology agents pose to widely-accepted methods used in early-phase clinical trials, and make recommendations for implementing innovative trial designs in future studies facing these challenges (Table 1).

**Table 1:** Design challenges, recommendations, assumptions, and available software for early-phase trials of imunne-oncology agents

Design Challenge	Design Recommendation	Endpoints	Model Assumptions	Available software
Late-onset DLTs	TITE-CRM [21] TITE-BOIN [23]	Binary toxicity	Probability of DLT increases with increasing dose level.	R Package dfcrm SAS macros https://sph.umich.edu/ccb/tite-resources.html www.trialdesign.org
Additional endpoints	Wages & Tait [30] Zang et al. [29] Ursino et al. [55]	Binary toxicity and binary efficacy (biologic activity or clinical response) Binary toxicity and continuous PK measures (AUC)	Probability of DLT increases with increasing dose level. Probability of efficacy increases or plateaus with increasing dose level. Toxicity related to the PK measure & PK triggers toxicity when above a certain threshold	https://uvatrapps.shinyapps.io/wtdesign/ www.trialdesign.org R Package dfpk
Drug combinations	POCRM [37] BOIN [39] PIPE [38]	Binary toxicity	Probability of DLT increases with increasing dose level of each agent for a fixed dose level of the other agent.	R Package pocrm www.trialdesign.org R package pipe.design
Dose and schedule	Braun et al. [52] Wages et al. [54]	Binary toxicity	Probability of DLT increases with increasing dose level of each agent for a fixed schedule.	https://biostatistics.mdanderson.org/softwaredownload/SingleSoftware.aspx?Software_Id=75 R Package pocrm

### Design challenges and recommendations

#### Late-onset toxicities

Dosing decisions in Phase I trials have traditionally been guided by DLTs that occur in cycle 1 of treatment, which is generally 28 days long. In these trials, MTDs are defined as the highest tolerated dose from cycle 1, even though patients are administered therapy over the course of several cycles. This approach was appropriate for cytotoxic chemotherapy agents, which generally cause DLTs to be observed early in the treatment course. However, acute toxicity does not provide a complete representation of tolerability for immune-oncology agents. These new agents are administered over extended periods of time, which can result in immune-related adverse events (irAEs) occurring outside of a short-term evaluation window. For example, in a pooled analysis of 576 patients with advanced melanoma who received nivolumab, the median onset time of various treatment-related adverse events of any grade ranged from 5.0 weeks for skin toxicities to 15.1 weeks for renal toxicities [15]. For patients treated with pembrolizumab, the median onset time of irAEs has varied from 1.3 months for hepatitis to 3.5 months for diarrhea [16]. Therefore, the assessment of appropriate doses based solely on DLT definitions from cycle 1 toxicity outcomes is insufficient for immune-oncology therapies. One possible solution to this limitation, which has been utilized in several recently published studies of immune-oncology agents [17–20], is to extend the DLT evaluation window. Additionally, to identify more appropriate doses for further research, there is a growing need to incorporate richer toxicity information beyond DLTs observed in cycle 1. The most well-known dose-finding method that allows for the incorporation of late toxicities into the design is the time-to-event continual reassessment method (TITE-CRM) [21]. This method utilizes information from partially observed subjects throughout the trial, without staggering enrollment. In the absence of a DLT, it weights each entered patient by the proportion of the full observation period that he/she has been observed. In the case of no DLTs outside the observation window, the method reduces to the original continual reassessment method (CRM) [22]. Given the availability of *R* packages (*dfcrm*) and SAS tools (https://sph.umich.edu/ccb/tite-resources.html) for simulating and conducting the TITE-CRM, the method can be easily tested and implemented into studies in which late-onset DLTs are anticipated. Another recently published method adapts the Bayesian Optimal Interval (BOIN) design to handle late-onset DLTs, and it is accompanied by a user-friendly web application at www.trialdesign.org [23].

#### More may not be better

The monotonicity assumption in the traditional Phase I setting is driven by the notion that cytotoxic chemotherapy treatments will directly inhibit the growth of malignant cells, and that the MTD will provide the greatest therapeutic benefit. Conversely, immune-oncology agents generally do not directly impact malignant cells. Instead, immune cells, such as T-cells or natural killer cells, indirectly facilitate the cytotoxic efficacy of these agents. The notion that “more is better” for efficacy and “more is worse” for toxicity may not hold true for immune-oncology agents, thus violating the monotonicity assumption that historically underlain Phase I trial designs. Consequently, the early development of immune-oncology agents may need to transition from identifying the MTD to identifying the minimum effective dose. In the case of a dose-efficacy curve that plateaus beyond a certain dose, the optimal dose may fall below the MTD and application of an MTD-based approach could lead to incorrect dosing. For example, in a study of nivolumab, no MTD was reached for doses of 1, 3, and 10 mg/kg using the original 3 + 3 design. However, based on initial signs of activity, additional expansion cohorts were added at doses far below the MTD (0.1 and 0.3 mg/kg), indicating a flat dose-efficacy curve among the dose being considered [5]. The dose-toxicity and dose-efficacy relationships for anti-PD-1/PD-L1 therapies remain unclear, with relatively flat toxicity and efficacy rates for doses ranging from 1 to 2 mg/kg to 20 mg/kg every 2 or 3 weeks [17, 18, 24]. Conversely, based on results from a study of ipilimumab, patients treated with 10 mg/kg demonstrated better overall survival than those treated with 3 mg/kg (15.7 months (95% confidence interval [CI] 11.6–17.8) versus 11.5 months (95% CI 9.9–13.3) (hazard ratio [HR] 0.84; *p* = 0.04)) [25]. The patients treated with 10 mg/kg also had a higher rate of irAEs than those treated with 3 mg/kg [20], indicating dose-dependent toxicity and efficacy relationships for ipilimumab. As for toxicity, early-phase data were extensively studied in a review of thirteen Phase I clinical trials of immune checkpoint blocking antibodies [26]. In this review, only one trial identified protocol-defined DLTs [27]. In most of the other trials, the RP2D was based upon the maximum administered dose, which in turn is based on a pre-specified dose range developed from pharmacokinetic data.

Implementation of novel approaches that incorporate multiple endpoints are needed to establish and refine the choice of recommended Phase II doses. In addition to a DLT endpoint, early-phase design strategies should incorporate a tumor activity endpoint, with the goal of identifying an effective dose [7, 28]. Activity markers may include early measures of efficacy (e.g., clinical response), altered pharmacokinetics, altered pharmacodynamic outcomes, and a persistent immune response. The field of immune-oncology often investigates treatments that demonstrate minimal overall toxicity, wherein higher doses may not induce a greater response. The treatment response may increase at low doses and then begin to plateau at higher doses. The goal of the trial then becomes identifying the optimal biologically active dose (OBD), defined as a safe dose that demonstrates the greatest pharmacological activity. In recent years, several new methods have been proposed for identifying the optimal safe and effective dose in Phase I-II trials [29, 30]. For the problem of locating the optimal biologic dose based on toxicity and activity endpoints in single-agent trials, web applications exist for simulation and implementation of the Wages and Tait [30] method at https://uvatrapps.shinyapps.io/wtdesign/ and for the Zang, Lee, and Yuan [29] method at www.trialdesign.org. One advantage of the Wages and Tait app is the ability of the user to incorporate a stopping rule that terminates the study once a pre-specified maximum number of patients have been accrued to one of the dose levels.

#### Drug combinations

It is becoming increasingly popular to treat patients with combination immunotherapy due to the potential for synergistic activity in which the efficacy of both agents together is higher than the efficacy of each agent alone, hopefully without significantly increasing toxicity. The selection of appropriate drug combinations for testing can be very challenging because single agent toxicity data may not be sufficient for characterizing the safety profile of the combination. The most effective and safest doses in drug combinations are seldom the same as those of the individual agents identified in monotherapy trials [31]. Drug combination dose-finding trials also present a greater challenge of finding an MTD combination, or combinations, due to the more complex toxicity and efficacy profiles presented by the potential interaction of the two agents. For example, it is recommended that, combinations of drugs with non-overlapping toxicity profiles be developed whenever possible, since overlapping toxicities can limit escalation of the combination doses to effective levels. In the case of non-overlapping toxicities, the DLT definition of the drug combination is specific to the agents being studied. In practice, Phase I drug combination studies necessitate significant planning at the design stage in order to establish the starting dose of each agent and the total number of combinations to be tested, and these studies can rapidly grow in sample size and cost [32].

In addition to the complexities mentioned above, drug combination studies present additional design challenges to those encountered in single agent studies. Because of the monotonicity assumption, single-agent trials are said to follow a complete order. This is because the ordering of DLT probabilities for any pair of doses is known, and administration of greater doses of the agent can be expected to produce DLT’s in increasing proportions of patients. The monotonicity assumption lends itself to escalation along a single line of doses. Given the toxicity response (DLT; yes/no) for a particular patient, either the same dose is recommended for the next patient or the dose is changed to one of two adjacent doses (i.e. either escalate to the next highest dose or de-escalate to the next lowest dose). In studies testing drug combinations, the probabilities of DLTs often follow a “partial order” meaning that there are pairs of combinations for which the ordering of the probabilities is unknown. In a multi-agent trial, there will most likely be more than one possible treatment with which to treat the next patient cohort in a decision of escalation, and it may not be clear as to which combination to the next cohort should receive.

A traditional approach to this combination dose-finding is to pre-select drug combinations with a known toxicity order and apply a single-agent design by escalating and de-escalating doses along a chosen path [33]. This could be done by, a priori, pre-specifying a subset of combinations for which the toxicity ordering is known. This approach transforms the two-dimensional dose-finding space into a one-dimensional space, and it has been used in much of the early work in dose combinations [34, 35]. The disadvantage of this approach is that it limits the number of dose combinations that can be considered and it can potentially miss promising dose combinations that exist outside of the path. More recent methods have moved away from reducing the two-dimensional dose-finding space to a single dimension, a thorough review of which has been written by Harrington et al. [36]. A number of designs have been proposed for finding the MTD of cytotoxic agents [37–39]. These methods determine combinations to which patients are allocated based solely on toxicity considerations, without accounting for efficacy. As in the single-agent setting, these model-based methods have superior performance to rule-based methods in terms of accuracy of MTD identification, and safety in allocating patients [32]. A web application for the Bayesian Optimal Interval (BOIN) method [39] for combinations is available at www.trialdesign.org, and *R* packages exist for the partial order continual reassessment method (package *pocrm*) [37] and the product of independent beta probabilities escalation (PIPE) design (package *pipe.design*) [38]. The POCRM was successfully implemented in a recently completed, but yet to be published, Phase I trial designed to determine the MTD of a combination of a toll-like receptor (TLR) agonists with or without a form of incomplete Freund’s adjuvant (IFA) for the treatment of melanoma (NCT01585350). To our knowledge, the PIPE design has been implemented in two dose-finding studies (NCT02760797, NCT02308072). There are a few existing early-phase designs for drug combination trials that account for both toxicity and efficacy. For example, the method of Wages and Conaway [40] has been adapted and implemented in recently completed and ongoing early-phase studies of combination immune-oncology agents (NCT02126579, NCT02425306) [41, 42] using immunologic response as a binary activity endpoint for driving the design. The *R* code used to successfully implement these designs are available at http://faculty.virginia.edu/model-based_dose-finding/.

The methods recommended in this section can broadly be implemented in early-phase combination studies that involve immunotherapies in combination with other immunotherapies, or in combination with chemotherapy, radiotherapy, or molecularly targeted agents. Each of these combination types may present their own specific set of trial design challenges, but the methodology described can be generally adapted and applied to meet the research objectives of a broad range of early-phase combination studies. As highlighted at the 2018 ASCO annual meeting, more work is needed in acquiring a better understanding of how to optimally combine therapies [43]. As we learn more, early-phase methodology may need to be adapted to handle unique design challenges associated with novel treatment combinations involving immunotherapies.

#### Dose and schedule

The lack of a clear dose-efficacy relationship for both anti-CTLA-4 and anti-PD-1 antibodies has resulted in these agents being assessed at various dose-schedule combinations. For example, ipilimumab was evaluated in four Phase I trials at doses ranging from 3 mg/kg to 20 mg/kg, without an MTD being identified in any of the trials. A subsequent Phase II trial compared three dose levels of ipilimumab in patients with metastatic melanoma (0.3, 3, and 10 mg/kg); this trial, along with a positive Phase III experience at 3 mg/kg, yielded the registration dose of 3 mg/kg for 4 cycles [44]. Concurrently, patients with resected melanoma were enrolled in a study using adjuvant ipilimumab at a higher dose (10 mg/kg) and with an alternative schedule (4 cycles every 3 weeks with maintenance doses every 3 months). This dose and schedule was FDA- approved in the adjuvant setting after it was shown to improve progression free survival [45].

Pembrolizumab has also been studied at different doses (2 mg/kg vs 10 mg/kg) and different schedules without a significant difference in efficacy or toxicity by dose or schedule [3, 46]. More recently, flat dosing of pembrolizumab at 200 mg every 3 weeks has been FDA approved for the treatment of squamous cell carcinoma of the head and neck and PD-L1 positive NSCLC [47, 48]. Further contributing to the uncertainty of dosing design is the use of alternative schedules when immune checkpoint inhibitors are used in combination. For example, the combination of nivolumab 1 mg/kg and ipilimumab 3 mg/kg dosed every 3 weeks for 4 doses is FDA-approved for the treatment of metastatic melanoma [49, 50]. Alternative dosing of the combination of nivolumab and ipilimumab was studied in a Phase I trial of patients with metastatic NSCLC in which patients were randomized to receive nivolumab 1 mg/kg every 2 weeks plus ipilimumab 1 mg/kg every 6 weeks, nivolumab 3 mg/kg every 2 weeks plus ipilimumab 1 mg/kg every 12 weeks, or nivolumab 3 mg/kg every 2 weeks plus ipilimumab 1 mg/kg every 6 weeks. Response rates and irAEs were similar in the two treatment groups that received nivolumab 3 mg/kg and both of these arms are considered promising for further study in the randomized Phase 3 trial Checkmate 227 [51].

For these trials, finding an acceptable dose and schedule becomes a two-dimensional dose-finding problem, wherein one dimension is the dose level of the agent and the other dimension is the schedule of therapy. In addressing this type of problem, the approach of Braun et al. [52] based on a time-to-toxicity endpoint, was used to design a dose and schedule finding study (NCT00350818) of de Lima et al. [53]. Available software can be accessed at https://biostatistics.mdanderson.org/softwaredownload/SingleSoftware.aspx?Software_Id=75. Wages, O’Quigley and Conaway [54] proposed a method for finding a maximum tolerated dose-schedule combination, based on a binary toxicity endpoint, and the *R* package (*pocrm)* can be applied to this setting.

### Further challenges

#### Novel endpoints in phase I trials

Under the new assumptions of milder, non-monotone toxicity profiles, determining the OBD is an attractive goal for the early-phase studies. Practically speaking, dose-finding studies incorporating multiple (biological) endpoints have become frequently used approaches for evaluation of targeted, non-cytotoxic drugs. However, there are several barriers that limit their potential to just exploratory endpoints. The inclusion of biological endpoints and determination of an optimal dose based on some biomarker occurrence should rely on pre-specified thresholds such as targeted plasma or blood drug concentration, or other immunologic parameter. Incorporating PK information in the dose finding process can provide a better estimation of the dose-toxicity curve while maintaining the performance in terms of MTD selection. However, in most phase I trials, the dose-finding and pharmacokinetics (PK) analyses are considered separately, which for small populations might impact the estimation of both the toxicity and PK parameters. Ursino et al. [55] developed and extended methods that take into account PK measurements in sequential Bayesian adaptive early-phase designs. Several models including PK measures either as a covariate or as a dependent variable are examined via simulations in terms of MTD percent correct selection (PCS) and the ability to estimate the dose-response curve. Operating characteristics are presented for a fixed sample size of 30 subjects, six pre-defined dose levels, and seven toxicity scenarios. The main conclusion is that good prior knowledge about PK can help reduce the percentage of overdosing without altering the MTD selection. Still, some of the methods presented (e.g., PKCRM) rely heavily on the choice of PK constraints which in some cases fail to achieve the true MTD. These adaptive pharmacokinetics-based dose-finding designs can be implemented using the *R* package (*dfpk*) [56]. Assessing pharmacodynamic (PD) markers as primary endpoints can also be challenging, as they not only require a strong scientific rationale, but also a non-invasive reproducible assay that can track PD markers with minimal harm to the patient [57]. Integration of clinical PK and pre-clinical PD has provided an additional modality of augmenting early clinical data with animal data, but nothing is relevant in the absence of definitive correlations between the target inhibition in PK or PD biomarkers and clinical efficacy (e.g., tumor response).

Adoptive T cell therapy is a rapidly emerging immunotherapeutic approach that consists of an infusion of genetically engineered T cells that express a specific antigen on their cell membrane. In 2017, based on a pivotal Phase II trial, the FDA approved the first chimeric antigen receptor (CAR-T) cell therapy (tisagenlecleucel) for children and young adults with B-cell ALL in a population with limited treatment and poor outcomes [58]. With 83% remission rate, this therapy has demonstrated early and durable response, but much remains to be learned regarding cell proliferation, persistence and mechanisms of relapse. An important predictor of the efficacy of CAR–T cells is their ability to expand in vivo in response to recognition of CD19^+^ target cells, and therefore, patients that failed to respond in prior studies typically had poor accumulation of CAR–T cells. Interestingly, a recent study investigating CD19 CAR–T cells demonstrated a correlation between cell dose levels (magnitudes of 10^5^ cells/kg), earlier/higher peak expansion and clinical response [59]. This finding was also seen in other studies that showed direct correlations between the number of transduced T cells and antitumor response [60], or correlation between clinical response and persistence of administered cells at one month [61]. Although CD19 CAR-T cells showed a therapeutic effect in patients with relapsed and refractory B-cell ALL, significant toxicities have occurred, especially after infusion of higher CAR-T cell doses. Data imply that an optimal dosing strategy to minimize toxicity would be to initially give low CAR-T cell dose to patients with higher tumor burden, whereas those with low tumor burden may require higher or repeated doses. Thus, under this paradigm shift, dose-finding trials driven solely by toxicity is no longer realistic. Early phase trials should start incorporating more immunological information, while still maintaining acceptable toxicity levels.

The majority of current trials include extended correlative studies, in order to identify promising biomarkers from investigation of immunologic factors of the tumor or tumor microenvironment. Immunologic characteristics within the peripheral blood may similarly help predict outcomes following immunotherapy and allow for immunologic monitoring (T-cell response or percent persistence of transduced T-cells) while on treatment. New dose-finding designs have proposed independent or joint modelling of toxicity and immunological outcomes, both in binary and continuous forms [62]. Also, multi-stage adaptive designs have become more frequent in the early stages of development, with patients being randomized towards doses with higher predicted efficacy. In the context of personalized medicine, immunotherapy is becoming more and more relevant, especially for establishing the patient’s immune system profile and developing a tailored treatment/scheduling regimen.

#### Expansion cohorts

Phase I trials often include a dose expansion phase with one or more dose expansion cohorts (DEC) after completion of dose escalation with an overall goal to further characterize toxicity, gain preliminary evidence of efficacy, and/or determine the RP2D. It has become common practice that Phase I studies of immuno-oncology agents include multiple DECs based on specific molecular characteristics, biomarker and/or disease type.

The goals of the DEC will drive the sample size of the cohort(s). If the objective is to gain a more precise estimate of the probability of toxicity as has been the conventional purpose of DEC, then expansion cohorts are based on a pre-specified number of patients (e.g., 5, 10, 15) treated at MTD. When less than 15 patients are targeted for each DEC, formal sample size justification may not be feasible. The resulting RP2D may differ from the MTD as additional toxicity data are gathered through the expansion phase. In one systematic review, among expansion cohorts with safety objectives, new toxicities were reported in 54% of trials and the R2PD was modified in 13% [63]. Re-evaluation of toxicity data after DECs is increasingly important for the safety assessment of checkpoint inhibitors given that adverse events can occur weeks to months after treatment. Approaches for incorporating the additional information include: combining the toxicity data from the initial dose escalation and DEC after all patients have been treated; re-evaluation of the MTD as data from the expansion cohort are obtained with safety stopping rules built-in; evaluation of the MTD incorporating both safety and efficacy; and evaluation of MTD for different sub-populations [64]. Simulation studies have demonstrated that failure to include toxicity outcomes from DECs can result in less accurate estimate of the MTD [63].

Given the advances in technology and in our understanding of tumor biology which have allowed for significantly more drug and drug combinations tested simultaneously, the need for DECs to assess antitumor activity earlier in the drug development has resulted in projected accruals of the DEC to mimic traditional Phase II sample sizes, per cohort [65, 66]. In this setting, when assessment of efficacy is the primary objective for the DEC, a formal sample size justification with power calculation is recommended along with pre-defined stopping rules for futility to avoid exposing a high number of patients to the risk of ineffective or potentially dangerous treatment.

However, justification of sample sizes can be difficult and has led to very large studies in immune oncology and not always a clear rationale upfront. The anti-PD-L1 compound avelumab is being studied in a Phase I trial with 16 expansion cohorts with a total projected enrollment of 1706 people. In 2011, Merck initiated a first-in-human trial to determine the safety and recommended dose of pembrolizumab in patients with advanced solid tumors (NCT01295827, NCT01772004) [3]. This Phase I trial ultimately enrolled more than 1200 patients. Impressive response rates and duration of response were observed in patients with metastatic melanoma and non–small cell lung cancer, resulting in the addition of DECs to assess efficacy in these two patient populations, evaluate alternative dosing regimens and candidate predictive biomarkers [67]. Although the 1000+ patient Phase I trial is not typical, inclusion of many DECs have become standard and in some ways replacing the traditional clinical trial sequence.

## Discussion

Clinical practice has rapidly incorporated immune-oncologic agents into the standard treatment and management of many cancers. In general, immunotherapy is a treatment modality that activates the immune system to eliminate cancer rather than attacking cancer cells directly. The clinical success of immunotherapy has challenged the existing paradigm for clinical research. With standard chemotherapy or molecularly targeted agents, clinical benefit usually occurs during active treatment and does not persist after treatment discontinuation unlike immunotherapy. Decades of rigorous evaluation through early and late phase clinical trials has informed the understanding and management of the short and long-term effects of toxicities from chemotherapy. However, less is known regarding the toxicities associated with immunotherapy.

Due to the activation of the immune system, immune-oncologic agents can inadvertently activate the immune system against self, resulting in significant immune-related adverse events [68]. Since serious immune-related adverse events represent immune activation, they may actually reflect therapeutic benefit. Furthermore, while low grade immune-related adverse events are managed with dose reductions, management of more severe adverse events includes administration of anti-inflammatory therapies such as corticosteroids, infliximab or mycophenolate to dampen immune activation [68, 69], potentially weakening therapeutic benefit.

Understanding the mechanisms of response and adverse events in the context of these agents is critical for the selection of appropriate clinical trial designs. According to the Institute for Clinical Immuno-Oncology between 2006 and 2014, the number of clinical trials registered on ClinicalTrials.gov increased from 9321 to 18,400 [70]. According to the “Medicines in Development for Immuno-Oncology 2017 Report,” from PhRMA in partnership with the American Cancer Society Action Network (ASCAN), more than 248 new immuno-oncologic agents are currently in clinical trials or awaiting U.S. FDA review [71]. In addition, from 2015 to 2017, the number of combination studies listed on ClinicalTrials.gov combining PD-1 or PD-L1 inhibitors with other therapies has more than tripled from 215 to 765 (combination trials with pembrolizumab [*n* = 268]; nivolumab [*n* = 242]; durvalumab [*n* = 123]; atezolizumab [*n* = 83]; avelumab [*n* = 18]; and others [*n* = 49]) [72]. Implementation of innovative design strategies in the early development of combination immunotherapies is critical to delivering more effective therapies with improved outcomes.

In this paper, we reviewed design considerations for early phase clinical trials of immuno-oncology agents and when available provided reference to software for applicability of these designs. There is increasing demand for study designs that are best suited and optimal in this setting. A deeper understanding of clinically meaningful endpoints, characterization of toxicity, identification of immune parameters and mutational burden to help guide patient selection will allow for the further development of novel designs for early phase trials of immuno-oncologic agents.

## Abbreviations

BOIN: Bayesian optimal interval design
CAR: Chimeric antigen receptor
CRM: Continual reassessment method
CTCAE: Common Terminology Criteria for Adverse Events
CTLA4: Cytotoxic T-lymphocyte antigen-4
DEC: Dose expansion cohort
DLT: Dose limiting toxicity
FDA: Food and Drug Administration
FDASIA: Food and Drug Administration Safety and Innovation Act (FDASIA)
IFA: Incomplete Freund’s adjuvant
irAEs: Immune-related adverse events
MTD: Maximum tolerated dose
NCI: National Cancer Institute
NSCLC: Non-small cell lung cancer
OBD: Optimal biologically active dose
PD: Pharmacodynamics
PD-1: Programmed cell death protein-1
PD-L1: Programmed death ligand-1
PIPE: Product of independent probabilities escalation
PK: Pharmacokinetic
POCRM: Partial order continual reassessment method
RP2D: recommended phase II dose
TITE-BOIN: Time-to-event Bayesian optimal interval design
TITE-CRM: Time to event continual reassessment method
TLR: Toll-like receptor
